# Reliability, validity and factorial structure of the Arabic version of the international suicide prevention trial (InterSePT) scale for suicidal thinking in schizophrenia patients in Doha, Qatar

**DOI:** 10.1186/s12888-016-1155-4

**Published:** 2016-12-07

**Authors:** Samer Hammoudeh, Suhaila Ghuloum, Ziyad Mahfoud, Mark Opler, Anzalee Khan, Arij Yehya, Abdulmoneim Abdulhakam, Azza Al-Mujalli, Yahya Hani, Reem Elsherbiny, Hassen Al-Amin

**Affiliations:** 1 Department of Research, Weill Cornell Medicine - Qatar, Doha, Qatar; 2 Department of Psychiatry, Rumailah Hospital, Hamad Medical Corporation, Doha, Qatar; 3 Department of Health Policy and Research, Weill Cornell Medicine - Qatar, Doha, Qatar; 4 CSO-Prophase, LLC, New York, USA; 5 Primary Health Care Corporation, Doha, Qatar; 6 Department of Psychiatry, Weill Cornell Medicine - Qatar, Education City, P.O. Box 24144, Doha, Qatar

**Keywords:** Suicidality, Suicide scales, Schizophrenia, Arabs

## Abstract

**Background:**

Patients with schizophrenia are known to have higher rates of mortality and morbidity when compared to the general population. Suicidality is a major contributor to increased mortality. The International Suicide Prevention Trial (InterSePT) Scale for Suicidal Thinking (ISST) is a validated tool to assess current suicidal ideation in patients with schizophrenia. The aims of the study were to culturally adapt the Arabic translation of ISST and to examine the psychometric characteristics of the Arabic version of the ISST among patients with schizophrenia in Qatar.

**Methods:**

ISST was translated and adapted into formal Arabic using the back translation method. Patients diagnosed with schizophrenia were randomly recruited from the department of Psychiatry, Rumailah Hospital, Doha, Qatar. Healthy controls were randomly recruited from two primary health care centers in Doha, Qatar. The Arabic version of Module B for suicidality in Mini International Neuropsychiatric Interview was used as the gold standard to which the Arabic ISST was compared.

**Results:**

The study sample (*n* = 199) was composed of 100 patients diagnosed with schizophrenia (age 35.30 ± 10.04 years; M/F is 2/1) and 99 controls (age 33.98 ± 8.33 years; M/F is 2/3). The mean score on the ISST was 3.03 ± 4.75 vs. 0.47 ± 1.44 for the schizophrenia and control groups, respectively. Inter-rater reliability coefficient was 0.95, *p* > 0.001. The overall Cronbach’s alpha was 0.92. Principal Component Analysis produced 3 factors explaining a total of 73.8% of variance.

**Conclusions:**

This is the first study in the Arab countries to validate the Arabic version of the ISST. The psychometric properties indicate that the Arabic ISST is a valid tool to assess the severity of suicidal ideation in Arabic patients with schizophrenia.

## Background

Schizophrenia is a chronic mental disorder characterized by debilitating positive and negative symptoms [1, 2]. The global burden of this disease can be conceptualized by a lifetime prevalence rate of 1% [3], and an incidence rate of 15.2 (median value) per 100,000 persons per year [4]. Furthermore, schizophrenia is associated with higher rates of morbidity and mortality when compared to the general population [5]. Hennekens et al. reported that patients with schizophrenia have a decreased life expectancy by 20% [6], where at least 40% of this premature mortality can be attributed to suicide and unnatural deaths [7].

A report by the National Association of State Mental Health Program Directors showed that schizophrenia patients have a 25 year mortality gap when compared to the general population [8]. About 30–40% of this premature mortality is due to suicide and injury and 60% due to medical reasons [8]. Palmer et al. reported a 4.9% lifetime suicide risk among schizophrenia patients [9]. Meltzer and Baldessarini showed that 9–13% of schizophrenia patients lose their lives due to suicide [10]. They further indicate that schizophrenia patients have a 0.2–0.3% annual suicide rate [10]. There are no systematic studies on suicidal behaviors in patients with schizophrenia in the Arab countries, probably due to the lack of validated instruments. A review on overall suicidality in the Arab countries by Karam et al. reported a lifetime suicide attempt rate of 0.72–6.3% and a suicide ideation rate of 2.09–13.9%, in the same population [11]. In the second part of the review, which included a larger number of Arab countries, the authors reported an annual suicide attempt rate of 1.9–127/100,000 in the general Arab populations [12].

Preston and Hansen in a review of suicide rating scales used in schizophrenia patients highlighted the importance of prevention of suicide [13], a process that starts by identifying the risk factors in the first place [14]. Such factors include delusions, auditory hallucinations, and poor treatment adherence [15]. Other studies point towards drug misuse and alcohol abuse, whether current or past [10, 16]. Depression [10, 17], history of suicide [10, 18, 19], history of hospitalization to prevent suicide [10], social isolation [18], and drug-induced parkinsonism [10] were reported by others as major risk factors in determining the possibility of future suicide attempts.

Several instruments have been designed to assess suicidality among mental health patients such as the Sheehan Suicidality Track Scale (Sheehan-STS) [20], the Beck Scale for Suicide Ideation (BSI) [21], the Positive and Negative Suicide Ideation Inventory (PANSI) [22], the Suicide Intent Scale (SIS) [23], the Columbia-Suicide Severity Rating Scale (C-SSRS) [24], and the Schizophrenia Suicide Risk Scale (SSRS) [25]. The International Suicide Prevention Trial (InterSePT) Scale for Suicidal Thinking (ISST) is the only scale specifically designed to assess current suicidal ideation among patients diagnosed with schizophrenia and schizoaffective disorder [26]. The ISST was initially developed in a study that assessed the effects of clozapine vs. olanzapine on preventing suicide among high risk patients diagnosed with schizophrenia and schizoaffective disorder [13, 27]. The ISST is composed of 12 items, and examines suicidal ideation along with its severity and related suicidal behavior [13]. The ISST takes approximately 20–30 min to be completed by clinicians, and each item is rated on a three level score system (0, 1, or 2) where the severity of suicidal thinking increases with higher numbers [26].

Lindenmayer et al. assessed the reliability and validity of the ISST among 1002 schizophrenia and schizoaffective patients, of which 22 patients had recent suicide attempt. The remaining sample (*n* = 980) had history of suicidal ideation in the past 3 years. For patients with recent attempts of suicide (*n* = 22), the results showed an intraclass correlation coefficient of 0.90 for the total ISST score and a mean weighted item kappa coefficients between 0.66–0.92. As for those patients who had suicidal ideation in the past 3 years (*n* = 980), the results showed a high internal consistency (Cronbach alpha = 0.86–0.89) for each of the items on the scale, and a Cronbach alpha coefficient of 0.88 for all items. The factor analysis on the ISST had three factors explaining 55.2% of the variance. Additionally, the results report a high correlation (*p* < 0.0001) between the Clinical Global Impression Scale-Severity (CGI-S) and the total score of the ISST. The ISST total score also significantly differentiated the various levels on the Clinical Global Impression Scale for Severity of Suicidality (CGI-SS) (*p* < 0.0001). The authors concluded that the ISST is a reliable and valid scale that can be employed by both researchers and clinicians, for suicidal risk assessment among patients with schizophrenia and schizoaffective disorder [26].

Currently, there are no standardized suicidality assessment scales in Arabic to assess suicide risk in Arab patients with schizophrenia. The objectives of this current study are to perform an Arabic translation and cultural adaptation of the ISST, and to assess its psychometric properties in terms of its validity, reliability and factor structure among patients with schizophrenia in Doha, Qatar.

## Methods

This study is part of a project which involved the Arabic translation, cultural adaptation and validation of several scales adopted in the assessment and treatment of schizophrenia, as the Positive and Negative Syndrome Scale (PANSS), the Calgary Depression Scale for Schizophrenia (CDSS), and ISST. The details reported here are those related mainly to the ISST.

### Translation and cross-cultural adaptation

A translation committee of three bilingual (English and Arabic) psychiatrists and a professional translator was formed. All psychiatrists have been practicing in Arab countries for over ten years (one trained in United Kingdom, one in United States of America and one in Egypt). These psychiatrists trained also the five raters who were involved in the administration of the Arabic ISST. The raters were supervised on at least five cases before they interviewed the participants alone. All raters were also bilingual (two physicians, two clinical psychologists and one nurse). The committee members independently translated the English version of ISST to Arabic and after deliberation they all approved one Arabic version of ISST. This version was first piloted in a sample of 20 subjects (10 schizophrenia and 10 controls). These subjects were interviewed individually. The most common theme was their reluctance to talk about suicide especially when discussing the suicide desire and the religious values. Most of the Arabs interviewed were Muslims and accordingly suicide is a taboo and religiously forbidden. This issue was addressed by training the raters to better engage the subjects and facilitate the disclosure on suicide. Few respondents commented that the Arabic translations of few words were not clear e.g “desire” (item 3), “passive” (item 4), “deterrants” (item 8), “contemplate” (items 9 and 10). These differences were attributed to the variations in common dialect in different Arabic subcultures. After the pilot phase, the psychiatrists and raters underwent three focus group sessions to review the comments and the raters’ experience with the pilot subjects. The raters commented mainly on the religious deterrants (item 8) and how this aspect might deter people from disclosing their thought about suicide. They also commented that formal Arabic language needed additional clarifications with the different Arabic subcultures. The raters added that the training helped them to decide on the rating of each item as there are no specific answers that can be given to subjects to choose from. The comments of the participants and raters in the pilot phase were reviewed again by the committee and further changes were incorporated in the second Arabic version that was sent for back translation by a new professional translator. The committee reviewed the back translation of the new professional translator and made minor modifications that were culturaly and clinically relevant. The final Arabic version, approved by all members of the committee, and its back translation to English were submitted then to the original author for revision. . The latter raised issues related to the back translation of few words to make sue that the concepts in each item are covered properly e.g item 1 (wish to die vs. desire to die), item 2 (reason vs. cause), item 4 (passive vs. inactive), item 10 (specificity vs. determination of method; considered vs. taken into consideration). The author had few clarifications and suggestions based on which the words were changed (in Arabic and back translation) before his final approval. The Arabic version of the corresponding approved back translation was then used to be validated in the Arabic subjects with schizophrenia.

### Participants

All participants signed a written consent form before joining the study. All study procedures and scales were explained to participants before signing the written consent. All study procedures complied with the most recent Declaration of Helsinki. Approvals from the Institutional Review Board (IRB) at Weill Cornell Medicine-Qatar (WCM-Q) as well as the IRB at Hamad Medical Corporation (HMC) were obtained before the initiation of study procedures. Patients diagnosed with schizophrenia (*n* = 100) were recruited from the Department of Psychiatry, Rumailah Hospital, HMC, Doha, Qatar. Healthy controls (*n* = 99) were recruited from two Primary Health Care Centers (PHCC) in Doha, Qatar. The Department of Psychiatry at Rumailah Hospital is the main psychiatric facility in Qatar. The ten outpatient clinics in the department receive approximately 120 patients on daily basis. The 4 inpatient wards have a 70 patient capacity, and are usually at 90–95% occupancy rate, where the length of stay is 2–4 weeks per patient. Medical records show that approximately 25% of the outpatients fulfill the criteria for the diagnosis of schizophrenia, while most of the inpatients get admitted with acute psychotic symptoms. The culture in Qatar is diverse with multiple ethnicities, languages, and traditions. Based on an official 2010 report, Qataris and Arabs (non Qatari) represent 15 and 13% of the population, respectively [28].

The inclusion criteria for patients with schizophrenia are: (a) between 18–65 years of age (b) diagnosed with schizophrenia as per the Diagnostic and Statistical Manual of Mental Disorders IV (DSM IV) and confirmed by the Mini International Neuropsychiatric Interview (MINI-6) Module K for schizophrenia and other psychotic disorders (c) speak Arabic as their first language (d) able to sign a consent form (e) patients must not have any comorbid medical conditions that could affect their ability to participate in the study. The exclusion criteria for the schizophrenia group are: (a) substance abuse over the last 6 months (b) impaired hearing or speech (c) high risk to harm self or others (d) presence of a psychiatric diagnosis based on DSM IV other than schizophrenia with or without depression. The inclusion and exclusion criteria for the controls are the same as those of the schizophrenia patients except that control subjects should not have schizophrenia or other psychiatric disorders except for depression. All subjects enrolled underwent a semi-structured interview to confirm the presence or absence of the diagnosis of schizophrenia (or other psychiatric disorders) using MINI-6 for schizophrenia and other psychotic disorders. The Arabic version of the MINI-6 has been previously used in several Arab countries to screen for mental disorders [29, 30].

In regard to sample size, we believe that we satisfied the condition of having at least 5 respondents per item [31] in a scale even if we use the PANSS as a reference, which has the largest number of items (30 questions) among the scales used. Furthermore, others reported that having about 200 subjects in a study to validate a scale is a fair number [32]. The sample was recruited using random sampling technique to try to give all the targeted subjects an equal chance of being recruited to the study. All patients with schizophrenia and all attendees at PHCC were randomly (every other patient) approached by their treatment teams to be enrolled in the study and only the eligible ones were selected if they accepted.

### Research design

The Arabic Module B for suicidality in MINI-6 was used as the gold standard to which the Arabic ISST was compared. Two blinded raters were involved in the validation process, the first administered the Arabic versions of the MINI-6 (modules B and K) and the CDSS, while the second administered the Arabic versions of the ISST and the PANSS. Questionnaires were also developed to obtain socio-demographic data and medical and psychiatric history. A total of 199 participants were recruited over a period of 14 months (September 2013 – Dececember 2015), among those, 42 cases were assessed independently by two raters for inter-rater reliability. These subjects were selected randomly based on the approval of subjects and the availability of two raters at the time of ISST assessment with the aim of having at least 40 subjects enrolled for this measure. The test-retest reliability was assessed using 22 cases who accepted to return after three days to be re-assessed by the same rater; the aim was to have at least 20 subjects for this measure.

### Statistical analysis

The Statistical Product and Service Solutions (SPSS) version 22 was used to run the different statistical analyses. Participants’ age and scores on the various instruments were summarized using means and standard deviations and were compared between the schizophrenia and control group using the independent *t*-test. The rest of demographic variables such as gender, marital status, etc. were summarized using frequency distributions and, whenever available, compared between the schizophrenia and control group using the Chi-squared test or Fisher’s exact test when expected cell counts fell below 5. A linear regression was carried out with the following sociodemographic variables (age, gender, country born, marital status, education and employment) along with the clinical characteristics (history of suicide and history of aggression) to explore their relation with ISST total score for patients with schizophrenia.

Internal consistency was assessed using Cronbach alpha. Inter-rater reliability was measured using the intraclass correlation coefficient (ICC). Test-retest reliability was also measured using the ICC along with the Pearson’s correlation between the total scores at baseline and the second measurement taken within 3 days. Correlation between ISST and MINI-6 Suicidality Module B scores were tested using Pearson’s correlation coefficient (*r*). Convergent validity was examined by comparing the ISST with item 8 on suicidality in the CDSS, using Pearson’s correlation. Discriminant validity was assessed by comparing the ISST to the different levels on Module B (no, low, moderate, or high risk of suicidality) of the MINI-6, using one-way analysis of variance (ANOVA). In addition, the ability of ISST to differentiate between patients’ with no or low suicide risk and those with moderate to high suicidality based on Suicidality Module B was assessed using Receiver Operating Characteristic (ROC) curve. Principal Component Analysis (PCA) with orthogonal rotation (varimax) was carried out to explore factor loadings, with a threshold of 0.50 [32]. Significance level for all tests was set at 0.05.

## Results

### Descriptives

The control group was composed of 99 individuals (mean age = 35.14 ± 10.08 years) (Table 1). The group of patients with schizophrenia was composed of 100 individuals (mean age = 33.96 ± 8.34 years). There were no age difference between the groups. However, there were significantly more males in the schizophrenia group and more females in the control group. The control group were more educated and the majority were employed when compared to the patients with schizophrenia (Table 1).

**Table 1:** Descriptive and clinical features of the patients with schizophrenia and healthy controls

	Schizophrenia group	Control group
	*N* = 100	*N* = 99
Age (Mean ± SD)	35.14 ± 10.08	33.96 ± 8.34
Gender*		
Male	67 (67%)	41 (41.4%)
Female	33 (33%)	58 (58.36%)
Country Born*		
Qatari	62 (62%)	35(35.4%)
Non-Qatari	38 (38%)	64 (64.6%)
Marital Status		
Married	16 (23.9%)	34 (82.9%)
Single	41 (61.2%)	7 (17.1%)
Divorced	9 (13.4%)	0 (0%)
Widowed	0 (0%)	0 (0%)
Missing	1 (1.5%)	0 (0%)
Education Level*		
Never Attended School	2 (2%)	0 (0%)
Intermediate / Elementary	35 (35%)	8 (8.1%)
Secondary / High School	39 (39%)	16 (16.2%)
Vocational Degree	2 (2%)	15 (15.2%)
College / Post-Graduate University	22 (22%)	60 (60.6%)
Employment*		
Employed / Student	40 (40%)	99 (100%)
Unemployed / Other	57 (57%)	0 (0%)
Retired	3 (3.0%)	0 (0%)
History of Aggression*	37 (37.0%)	2 (2.0%)
History of suicide attempts	30 (30%)	0 (0%)
Frequency of suicide attempts (median/range)	2 (1–7)	0 (0%)
ISST total score(Mean ± SD) *	2.94 ± 4.64	0.47 ± 1.44
Module B total score *	5.54 ± 12.63	0.90 ± 4.99
PANSS total score (Mean ± SD) *	73.11 ± 23.10	31.59 ± 7.52
CDSS total score (Mean ± SD) *	4.28 ± 4.33	1.35 ± 2.70

Significance based on Chi square for categorical variables and *t*-test for continuous variables

*SD* Standard Deviation

*p < 0.05

Patients with schizophrenia in this study reported having history of aggression significantly more than control subjects. In addition, none of the individuals in the control group reported having attempted suicide. In the patients with schizophrenia group, a total of 30.3% had a history of suicide attempts where the number of attempts ranged from 1–7 over the course of their illness. The mean total scores on ISST, Module B, CDSS and PANSS were significantly higher in patients with schizophrenia compared to individuals in the control group (Table 1). In the schizophrenia group, the mean age of first psychiatric diagnosis was 23.64 ± 7.76 years, while age of onset of symptoms was 22.88 ± 8.29 years. The median number of hospitalizations was 3 (range 1–18).

Linear regression for the different sociodemographic variables and clinical characteristics was significant, F (8,78) = 3.939, *p* = 0.001. All the sociodemographic variables were not significant predictors of ISST total score. However, having a history of suicide attempt or aggression did predict a higher score on ISST (*p* < 0.05).

### Reliability

The mean ISST score for schizophrenia patients in this study was 3.03 ± 4.75, with items means ranging from 0.12 to 0.41. Overall Cronbach alpha was 0.92, which is considered as high level of internal reliability [33]. The latter remained above 0.90 if any of the items were deleted from the analysis; hence none of the items were dropped (Table 2). The ICC for the inter-rater reliability including active and control subjects (*n* = 42; n represents the number of patients and healthy control cases that had the ISST administered by two independent raters) ranged between 0.79 and 0.97 for the ISST items and was 0.96, *p* < 0.001 for the total ISST score (Table 3). Taking schizophrenia group alone (*n* = 22) the ICC for the total ISST score was 0.95, *p* < 0.001. According to Cicchetti, these inter-rater reliabilities for the total ISST score are excellent [34]. Test-retest reliability (*n* = 22 subjects with schizophrenia; n represents the number of cases that had completed ISST twice within 3 days) was also high with ICC of 0.93, *p* < 0.001 and *r* = 0.56, *p* < 0.001. Such measures for the test-retest reliability are considered good [35].

**Table 2:** Psychometrics properties and factor structure of the 12 item ISST among patients with schizophrenia only (*N* = 100)

	Factor loadings					
	Current suicidal thinking	Passive suicidal ideation	Suicide contem-plation	Mean	SD	Cronbach’s alpha if item deleted
Q1. Wish to die	0.72			0.41	0.68	0.91
Q3. Desire to make active suicide attempt	0.84			0.24	0.54	0.91
Q7. Control over suicidal action/acting out/delusions/ hallucinations or self-harm	0.81			0.24	0.54	0.91
Q8. Deterrents to active attempt	0.74			0.25	0.59	0.91
Q9. Reasons for contemplating attempt	0.72			0.32	0.68	0.92
Q11. Expectancy/anticipation by patient of actual attempt	0.73			0.21	0.55	0.91
Q12. Delusions/hallucinations of self-harm	0.80			0.21	0.51	0.91
Q4. Passive suicidal desire		0.80		0.23	0.47	0.92
Q5. Frequency of suicidal ideation		0.83		0.20	0.45	0.92
Q6. Attitude towards ideation/wish		0.76		0.19	0.45	0.91
Q2. Reason for living vs dying			0.92	0.42	0.62	0.93
Q10. Method: Specificity/planning of contemplated attempt			0.56	0.12	0.33	0.92
Cronbach’s alpha	0.92	0.82	0.48			
Eigenvalues	6.700	1.119	1.037			
Percentage of Variance explained	55.83%	9.33%	8.64%			

Kaiser-Mayer-Olkins Sampling of Adequacy KMO = 0.837. Barttlet’s test, *χ* ^2^ (66) = 795.64, *p* < 0.001

**Table 3:** Intraclass correlation coefficient showing inter-rater reliability for each Arabic ISST item*

	ICC^*^	95% CI
Wish to die	0.93	(0.88, 0.96)
Reason for living vs. dying	0.88	(0,78, 0.94)
Desire to make active suicide attempt	0.97	(0.94, 0.98)
Passive suicidal desire	0.80	(0.63, 0.89)
Frequency of suicidal ideation	0.92	(0.85, 0.96)
Attitude towards ideation/wish	0.90	(0.82, 0.95)
Control over suicidal action/acting out/delusions/ hallucinations or self-harm	0.86	(0.74, 0.92)
Deterrents to active attempt	0.84	(0.69, 0.91)
Reasons for contemplating attempt	0.97	(0.95, 0.99)
Method: Specificity/planning of contemplated attempt	0.79	(0.59, 0.89)
Expectancy/anticipation by patient of actual attempt	0.90	(0.82, 0.95)
Delusions/hallucinations of self-harm	0.97	(0.95, 0.99)
InterSePT Total Score	0.96	(0.92, 0.98)

*CI* Confidence Interval

^*^Sample size for inter-rater reliability was 42 (22 schizophrenia and 20 controls), *p* < 0.001

### Consruct validity: PCA

The PCA produced 3 factors explaining 55.83%, 9.33%, and 8.64% percent of the variance (total 73.8%) respectively [36]. Factor 1; labeled as current suicidal thinking, included items # 1, 3, 7, 8, 9, 11, and 12. Loadings on this factor ranged from 0.72–0.85 (Table 2). Factor 2; labeled as passive suicidal ideation, included items 4, 5, and 6. Loadings on this factor ranged from 0.76–0.83. Factor 3; labeled as suicide contemplation, included items 2 and 10. Loadings on this factor were 0.92 and 0.56, respectively.

### Other validity measures

#### Concurrent validity

ISST total score significantly correlated with the scores on MINI-6 Suicidality Module B, *r* = 0.62, *p* < 0.001 (Table 4).

**Table 4:** Concurrent, discriminant and convergent validity measures of the Arabic ISST

	Statistical test		95% CI	*p*-value
Concurrent Validity: ISST total score with Module B total score	Pearson’s Correlation	0.64	(0.43, 0.78)	*p* < 0.001
Discriminant Validity:				
ISST total score with Module B suicidality risk:	ANOVA			
None (reference group)	EMM	0.59	(0.12, 1.05)	
Low	EMM	2.71	(1.85, 3.57)	*P* = 0.004^a^
Moderate	EMM	6.57	(4.50, 8.65)	*P* = 0.28
High	EMM	9.67	(7.83, 1.50)	*P* = 0.01^a^
Convergent Validity:				
ISST total score with CDSS total score	Pearson’s Correlation	0.61	(0.42, 0.73)	*p* < 0.001
ISST total score with PANSS total score	Pearson’s Correlation	0.43	(0.29, 0.57)	*p* < 0.001

^a^Compared to the reference group with no suicidality. EMM: Estimated Marginal Mean of ISST total score. Sample used in the validity tests includes both groups, schizophrenia and control groups and the sample size is 199

#### Discriminant validity

**Fig. 1:**
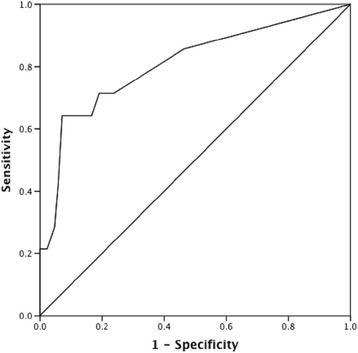
Receiver operating characteristic (ROC) curve for the total ISST scores obtained from the schizophrenia group (*N* = 100) normalized to the total score on MINI-6 Module B that was used to separate patients with no or low risk vs. those with moderate to high suicidality risk. Area under the ROC curve (AUC) is 0.808 with 95% CI (0.669–0.948)

The total Arabic ISST score differentiated between the different levels of suicidality according to module B. ANOVA showed significant level effect, *F* (3, 193) = 39.52, *p* < 0.001. The difference according to Games Howell post hoc test was significant between the level of no suicidality based on module B and those belonging to the low and high, *p* < 0.001 [37]. In addition, the ISST was also able to differentiate between those with no or low suicide risk and those with moderate to high suicidality based on module B, with a sensitivity of 71.4% and a specificity of 81%. The area under the curve was good (0.81) indicating that using the ISST score one can accurately classify 81% of the patients into one of two groups (no/low vs. moderate/high suicidality) (Fig. 1).

#### Convergent validity

The correlation of the total ISST score with the item on suicidality in CDSS was significant, *r* = 0.67, *p* < 0.001. The correlation of ISST total score with that of PANSS was also significant but of lower magnitude (*r* = 0.43, *p* < 0.001).

## Discussion

This study aimed at investigating the psychometric properties of the Arabic version of the ISST in a sample of Arabic patients with schizophrenia and controls in Doha, Qatar. The results showed that the Arabic version of the ISST had satisfactory psychometric properties with good reliability and validity measures. The Arabic ISST score can also help to differentiate those with no or low suicidality risk vs. those with moderate to high risk. This is the first study to validate the Arabic ISST and as it was done in formal Arabic, the official language in all Arab countries, it can be widely used by all Arabs to assess suicide in patients with schizophrenia. This study have many strengths e.g the adoption of standard methods for translation and cultural adaptation of scales, the inclusion of a control group to confirm the specificity of ISST for patients with schizophrenia, and the training of the raters who were blind to the scores of the gold standard.

Overall internal consistency was adequate (Cronbach alpha = 0.92) and remained over 0.90 if any item was dropped. This means that every item in the Arabic ISST is well related to the total ISST score and that each one is contributing to the general construct of the scale. Inter-rater reliability was good for the total ISST score (ICC = 0.96) and individual items. However, items 2 (reason for living vs. dying), 4 (passive suicidal desire), 7 (control over suicidal action/acting out/delusions/hallucinations or self-harm), 8 (deterrents to active attempt), and 10 (method: specificity/planning of contemplated attempt) had a lower ICC (0.79–0.88). These lower values are probably related to the variability in the raters’ ability to engage subjects and to facilitate the disclosure about history of suicidal ideation and attempts. The reliability measures of the Arabic ISST are similar to those reported for the original English ISST [26] and Polish version [38] in regard to internal consistency and inter-rater reliability. This is the first study to report on test retest reliability with ISST. The results of the PCA (construct validity) produced also three components as the English [26] and Polish ISST [38]. In the Arabic ISST, the first factor involved suicidal thinking in the current state, the second one covered passive suicidal ideation, and the third factor involved suicide contemplation. However, the validation study with the original ISST revealed a total variance of 55.2% for the three factors, which is less than the variance produced for the Arabic ISST (73.3%). In addition, the loadings on each factor were also different in the original ISST (36.4% factor 1: current suicidal thinking; 9.4% factor 2: volitional suicidal thinking; 8.4% factor 3: cause of suicidal thinking). The first factor in both studies involved current suicidal thinking and mutually included items # 1 and 3. The second factor mutually included items # 4 and 6. While the third factor included no mutual items. The differences in the factor structure in both studies are probably due to several methodological and cultural differences. For example, in the original validation study the sample size was larger and all subjects had schizophrenia with history of suicidal ideation or attempts, while in our study many schizophrenia patients had no such history. Furthermore, most of our subjects were reluctant to elaborate on the history of suicidality for religious reasons as presented above in relation to cross-culural issues. The validation study of the Polish version of the ISST reported that the PCA produced also three factors, which were reported to be congruent with those of the English ISST [26]. However no details are available as only an abstract [38] was published on the Polish version.

The total score of the Arabic ISST significantly correlated with the score of the Arabic MINI-6 Suicidality Module B, the only available Arabic gold standard to assess suicidality. The score was also able to differentiate patients with no/low suicidality risk from those with moderate/high risk with adequate sensitivity and specificity. Similar to the results of the English ISST [26], the convergent validity of the Arabic ISST was also significant when the total score on the ISST was compared to the item on suicidality in the Arabic CDSS and the total score of the Arabic PANSS. Ayer et al. examined the ability of the ISST to predict attempts or hospitalizations to prevent attempts of suicide in a study that compared the ISST to the results of the CDSS. The study concluded that higher numbers on both scales might be a useful indication as to when preventive measures need to be considered [39].

The design of this study have several strengths that were presented above, however there are few limitations that are worth discussing. First, module B inquires about suicidality over the past month, while ISST covers the suicidal ideation in the past week. This would possibly produce some variability that will not be assessed by the different validity measures. However, the two scales significantly correlated in our study. Second, ISST is specifically designed for schizophrenia patients while MINI-6 Module B is a tool to assess suicide in general, and thus the latter might not capture the command hallucinations and their delusional interpretation on the risk of suicide in patients with schizophrenia. However, the two Arabic tools correlated significantly in this study. Third, the sample had more males and there might a gender that limits the generalizability of the results. This gender bias is probably due to the nature of the disease being more severe among males and many of the schizophrenia patients were recruited from the inpatient wards. Another contributing factor to this bias is the population distribution in the country being skewed towards males where the male/female ratio is 3 [28]. Finally, both scales use formal Arabic which is the official language in all Arab countries but it is not the common dialect that is used daily by most Arab people; and thus its understanding might be confounded by the socioeconomic status and level of education of the Arab participants enrolled in this study.

## Conclusions

This study provides a culturally adapted and scientifically validated Arabic ISST that can be used in the assessment of suicidality among Arab speaking patients with schizophrenia. However, larger studies are needed to include more Arab people from the various Arabic cultures to check for possible confounding factors like nationality and level of education, and to check if our findings are generalizable to all Arab countries.

## Abbreviations

ANOVA: Analysis of variance
BSI: Beck scale for suicidal ideation
CDSS: Calgary depression scale for schizophrenia
CGI-S: Clinical global impression scale-severity
CGI-SS: Clinical global impression scale for severity of suicidality
CI: Confidence interval
C-SSRS: Columbia-suicide severity rating scale
DSM-IV: Diagnostic and statistical manual of mental disorders IV
EMM: Estimated marginal mean
HMC: Hamad medical corporation
ICC: Intraclass correlation coefficient
InterSePT: International suicide prevention trial
IRB: Institutional review board
ISST: International scale for suicidal thinking
MINI-6: The mini international neuropsychiatric interview-6
PANSI: Positive and negative suicide ideation inventory
PANSS: Positive and negative syndrome scale
PCA: Principal component analysis
PHCC: Primary health care corporation
QNRF: Qatar national research fund
QSA: Qatar statistical authority
ROC: Receiver operating characteristic
SD: Standard deviation
Sheehan-STS: Sheehan suicidality track scale
SIS: Suicide intent scale
SPSS: Statistical product and services solutions
SSRS: Schizophrenia suicide risk scale
WCM-Q: Weill Cornell Medicine-Qatar
